# How Retroviruses and Retrotransposons in Our Genome May Contribute to Autoimmunity in Rheumatological Conditions

**DOI:** 10.3389/fimmu.2020.593891

**Published:** 2020-11-13

**Authors:** Tomas Mustelin, Kennedy C. Ukadike

**Affiliations:** Division of Rheumatology, Department of Medicine, University of Washington, Seattle, WA, United States

**Keywords:** autoimmunity, retrotransposons, retroelements, nucleic acid sensors, reverse transcriptase, type I interferon, endogenous retroviruses

## Abstract

More than 200 human disorders include various manifestations of autoimmunity. The molecular events that lead to these diseases are still incompletely understood and their causes remain largely unknown. Numerous potential triggers of autoimmunity have been proposed over the years, but very few of them have been conclusively confirmed or firmly refuted. Viruses have topped the lists of suspects for decades, and it seems that many viruses, including those of the Herpesviridae family, indeed can influence disease initiation and/or promote exacerbations by a number of mechanisms that include prolonged anti-viral immunity, immune subverting factors, and mechanisms, and perhaps “molecular mimicry”. However, no specific virus has yet been established as being truly causative. Here, we discuss a different, but perhaps mechanistically related possibility, namely that retrotransposons or retroviruses that infected us in the past and left a lasting copy of themselves in our genome still can provoke an escalating immune response that leads to autoimmune disease. Many of these loci still encode for retroviral proteins that have retained some, or all, of their original functions. Importantly, these endogenous proviruses cannot be eliminated by the immune system the way it can eliminate exogenous viruses. Hence, if not properly controlled, they may drive a frustrated and escalating chronic, or episodic, immune response to the point of a frank autoimmune disorder. Here, we discuss the evidence and the proposed mechanisms, and assess the therapeutic options that emerge from the current understanding of this field.

## Relevant Molecular Concepts of Human Autoimmune Diseases

An important feature of clinical autoimmunity is that patients tend to fall into a discrete number of reasonably well delineated and named disease entities (i.e. diagnoses), rather than spanning the full spectrum of autoimmunity against random antigens. Granted, there is variability and heterogeneity within most such disease entities; and some diagnoses may in fact represent more than one distinct disease or a series of mechanistically different molecular “endotypes” of the disease. Nevertheless, patients that deviate radically from the typical disease profiles are rare. This pattern of many distinct diseases does not readily mesh with the commonly accepted notion that autoimmunity starts with a simple stochastic loss-of-tolerance blunder by a T cell. Rather, it seems that autoimmune diseases must be the result of distinct and unique pathophysiological processes that evolve over an extended period of time into a specific disease. The two concepts are of course not mutually exclusive, but they shape our thinking in different ways: while the former focuses autoimmunity research on T or B cell antigen receptor repertoire and mechanisms of central and peripheral tolerance, the notion that autoimmunity may arise from specific biological processes broadens the search for disease triggers and attempts to understand the escalation towards disease. The therapeutic ramifications of these two views are also distinct: a T or B cell-centric view calls for immunosuppressive or tolerizing approaches, while the concept of specific biological processes resulting in autoimmunity will look for specific modulation of such processes without the need for suppressing the normal function of the immune system. In this review, we follow the notion that autoimmune disease can have causes other than “stochastic mistakes of adaptive immunity”. We accept that T and B cells are critically important for autoimmunity, but we are not convinced that they initiate it.

Although individual autoimmune diseases can be clinically quite different from each other and often are associated with polymorphisms in different genes, and may respond to different targeted therapies, it is also clear that some diseases likely have overlapping pathogenic mechanisms; these mechanistically “related” diseases share cardinal features and symptoms and can co-occur in individual patients (*e.g.*, SLE and secondary Sjögren’s syndrome). An example of a group of such “related” autoimmune disease are those characterized by elevated type I interferons (IFNs) (1–4), including large portions of systemic lupus erythematosus (SLE) (1, 5, 6), dermatomyositis (DM) (7), primary Sjögren’s syndrome (pSS) (8–10), and several others. Type I IFNs are a hallmark of anti-viral immunity, with which these diseases appear to share other features as well, including autoimmunity against a similar set of proteins involved in nucleic acid processing, as well as the nucleic acids themselves. As in autoimmune diseases, viral infections are often accompanied by fever, headache, loss of appetite, malaise, fatigue, arthralgias, and sometimes skin rash. During viral infections, these responses are transient, while in SLE they become chronic with an unpredictable and often episodic course.

We recently reviewed the currently known and proposed sources of pathogenic nucleic acids and how they can act to drive SLE-like autoimmunity (11). Briefly, the offending nucleic acid could be either DNA or RNA, or both. Pathogenic cytosolic DNA may leak out from the nucleus following extensive damage to chromosomal DNA or mitotic catastrophes (not very likely in autoimmunity). DNA can escape from defective mitochondria, or DNA can be synthesized by reverse-transcription of various species of RNA (particularly from retroelements). Extracellular DNA may spill out from cells dying by a variety of programmed cell death mechanisms, or from commensal gut microbes, and then be internalized and sensed by immune cells. Pathogenic RNA can be (mis)generated and sensed intracellularly or end up in the extracellular space from which it can be internalized, for example as part of immune complexes, to be sensed by endosomal toll-like receptors (TLRs) in immune cells. In this review, we focus on RNA transcripts derived from endogenous retroviruses and retrotransposons and on the extrachromosomal DNA synthesized by reverse transcription of these RNA species. We also discuss the potential contributions of proteins generated by translation of these RNA transcripts, which may form more or less complete virions.

An important concept to keep in mind when contemplating how aberrant DNA or RNA drive autoimmunity is that a multitude of ancient and powerful mechanisms exist within our cells to effectively prevent the expression of potentially problematic sequences in our genome and to effectively degrade and remove aberrant DNA or RNA. These mechanisms are reviewed in section *Defense Mechanisms Against Retroviruses and Retrotransposons: Our Original Immunity*. Their importance to our health is perhaps best illustrated by the serious diseases that arise from mutations in the genes for several of these pathways, including Aicardi-Goutières Syndrome (AGS), which is characterized by constitutively elevated type I interferons and SLE-like autoimmunity. It presents at birth as a suspected neonatal viral infection, which is a medical emergency, but no exogenous virus can be found and the disease continues unabated. Over the years, AGS patients develop severe neurological deficits, perhaps due to direct neurotoxicity of type I IFNs. In regular polygenic SLE, however, it remains unclear if these defense mechanisms are weakened or simply overcome by an abundance of aberrant RNA or DNA. There are many potential variants of these scenarios. We have proposed that the clinical heterogeneity of SLE may be due, in part, to heterogeneity in which pathogenic nucleic acid molecules are present and which sensors and pathways they trigger in individual patients (11). Elucidation of these events may result in the recognition of distinct “endotypes” of SLE, each with its specific therapeutic opportunities.

## Endogenous Retroviruses and Retroelements in Our Genome

“By DNA sequence, we are more retroviral than human” is a provocative way of pointing out that a considerably larger portion of our human genome consists of sequences that once were RNA genomes of free and infectious retroviruses that were reverse transcribed into DNA and then pasted into our genome; they are more abundant (8%) than all the exons of our protein-coding “traditional” genes combined (about 1%) (12). Since most, if not all, genomes of eukaryotic and prokaryotic organisms on our planet share this feature of abundant inserted retroviral sequences, it is very likely that the 8% of our genome that is readily recognizable today as retroviral in origin is, in fact, only the tip of the iceberg. Most such sequences are not positively selected for (but likely the opposite) and over evolutionary time lose their integrity by random mutations, deletions, recombinations, and other mechanisms. For these reasons, sequences older than 100 million years become increasingly difficult to recognize with acceptable confidence.

**Figure 1:**
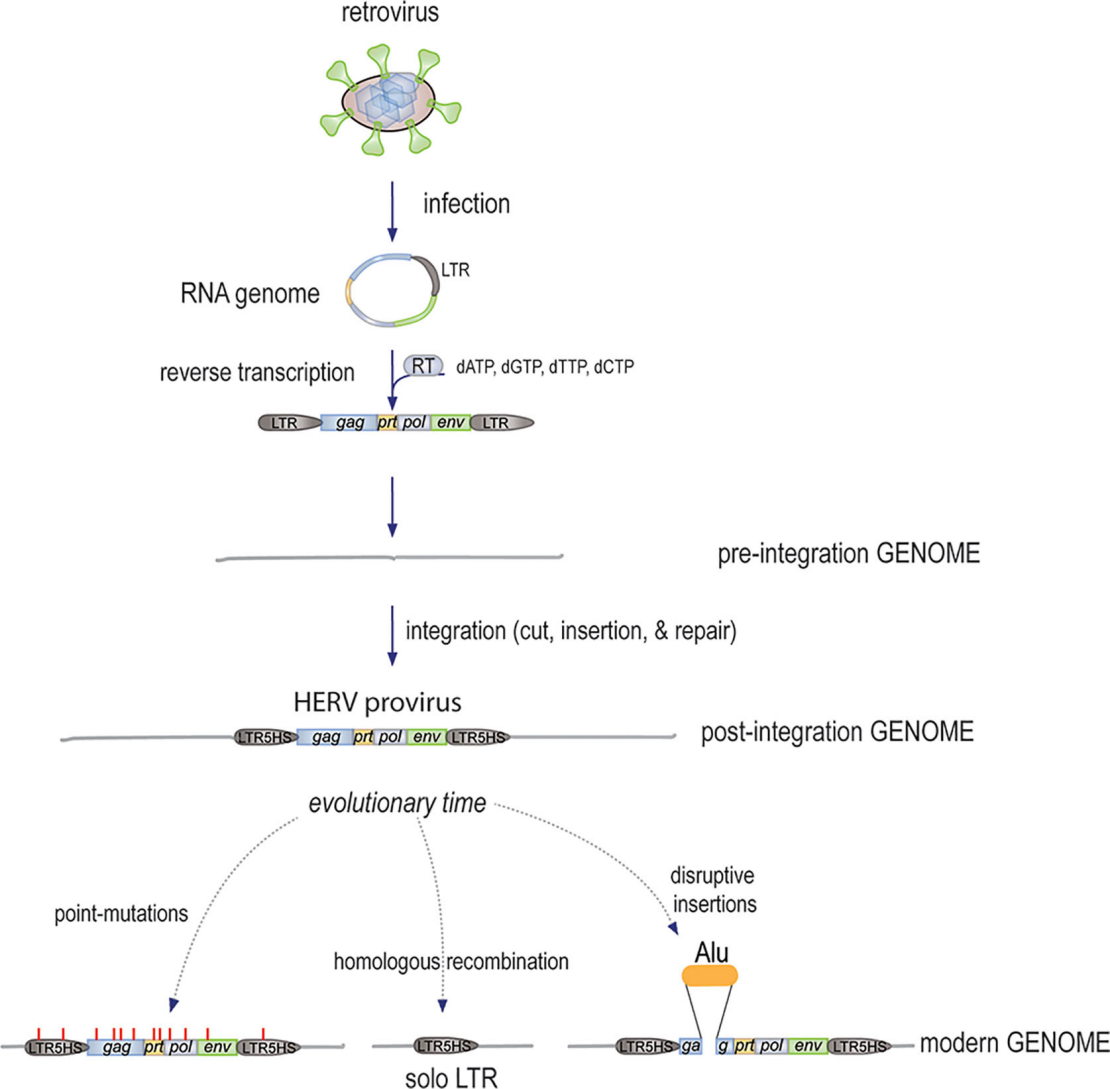
The mechanism by which new HERVs were generated. An infectious free retrovirus infects a host germline cell, releases its circular RNA genome, which remains protected by the associated *gag*-derived nucleocapsid and other core proteins, while the *pol*-encoded RT synthesizes the first strand of linear cDNA starting with the LTR and ending after the second copy of the LTR, followed by second strand synthesis. The resulting dsDNA of approximately 9,500 bp is then inserted into the genome by the endonuclease activity of the Pol protein. The ends are then finalized by the DNA repair machinery with a few added nucleotides. Over evolutionary times, further changes to the HERVs included the accumulation of point-mutations (some introducing stop-codons), deletions by homologous recombination, and disruption by additional insertions.

Since reverse-transcribed retroviral sequences are present in all kingdoms of life, it appears that this influx of genomic material started at the very dawn of cellular life (13–16). In fact, it is quite likely that it was instrumental to the evolution of larger and more diverse genomes: each newly incorporated reverse-transcribed retroviral genome adds ~ 9,500 base pairs to the genome (**Figure 1**), including three major protein-coding genes, *gag*, *pol*, and *env*, plus mRNA splicing sites, to generate transcripts that are translated into at least five proteins, each of which can be proteolytically processed into additional functional units. In addition, each retroviral integration brings two identical long terminal repeats (LTRs), one on each end of the insert, which contain clusters of highly efficient transcription factor binding sites to control transcription of the insert, as well as adjacent regions. In fact, it has been estimated that more than 300,000 regulatory regions (including promoters and enhancers) in our genome are, or contain remnants of, ancient LTRs from otherwise long-lost retroviral inserts. It is also clear that many “traditional” genes are descendants of ancient retroviral *gag*, *pol*, or *env* genes that were co-opted for new uses (17). For example, the RNaseH and integrase domains of the retroviral *pol* gene served as starting material for fundamental building blocks of our immune system (18). It has also been suggested that mRNA splicing was originally a retroviral invention.

The 8% of our human genome that consists of recognizable proviruses (12) (*i.e.* the cDNA of retrovirus RNA genomes, or parts of them), are collectively termed the human endogenous retroviruses (HERVs). Strictly speaking, this term is not entirely accurate since the majority of these sequences were incorporated long before our hominin ancestors became *Homo sapiens*. Hence, the term HERVs should be viewed as the complement of retroviral sequences in their current state, which for essentially all of them is different from what they looked like when they were free and contagious retroviruses that infected our ancestors and in the process reverse-transcribed their RNA genome and inserted it into the germline of their host. Although they were subsequently inherited in a Mendelian fashion by all descendants of the original host, there was little evolutionary pressure to maintain them in their intact form; more likely the opposite. Chimpanzees and gorillas have remarkably similar sets of retroviral loci (19–21), except for different mutations and the dozen or so new integrations that occurred in each species since our last shared ancestor lived approximately 6 million years ago.

In addition to the *bona fide* HERVs, an even larger portion of our genome, over 30%, consists of copies of non-LTR retrotransposons (12). Collectively, endogenous retroviruses and retrotransposons are referred to as retroelements. The non-LTR retrotransposons are classified as either “autonomous” or “non-autonomous” depending on whether they contain all the required components necessary for retrotransposition within themselves, or not. The most abundant class of autonomous retrotransposons are the long interspersed nuclear elements-1 (LINE-1 or L1 for short) (22). The biology and potential relevance of L1 in autoimmunity is discussed in section *Non-LTR Retrotransposons—LINE-1 and Alu Elements*.

The non-autonomous retrotransposons include the short interspersed nuclear elements (SINE), such as the Alu (23) elements and SINE-R, VTR, Alu (SVA) elements (24), which all depend on the L1 reverse transcriptase (RT) for their retrotransposition cycle. The Alu sequence itself appears to be a contracted form of the 7SL RNA (25), which is a component of the signal recognition particle. Alu elements have been extraordinarily successful in replicating within our genome: there are approximately 1 million of them in our genome, many of them within introns, where they may modulate gene transcription and mRNA processing.

### The Many Families of Retroviral Sequences in Our Genome

The tens of thousands of retroviral sequences that exist in our genome belong to more than a dozen distinct families, which originally were distinct free retroviruses that infected our ancestors during different, but often overlapping, epochs of prehistorical times. Most of these sequences have accumulated numerous mutations and deletions, and some have been disrupted by insertions of other retrotransposons, e.g. Alu elements. The older sequences have more such alterations and have lost their ability to encode full-length retroviral proteins, but the more recently incorporated ones are more complete and still retain the capacity to encode fully functional proteins and to produce viral particles. However, it seems that none of the HERVs are fully infectious anymore.

A basic nomenclature divides Retroviridae into four classes: gammaretroviruses (class I), betaretroviruses (class II), spumaretroviruses (class III), and lentiviruses (class IV). The first three classes are represented in our genome. They are further divided based on the specific tRNA they use for priming of reverse transcription. In essence, the retroviral RT that generates a DNA copy of the circular viral RNA genome uses a cellular tRNA complementary to a short motif in the viral LTR for priming of the reaction. The youngest of the Class II HERVs, for example, used lysine-tRNA for priming and are therefore classified as group K (for lysine), hence named HERV-K. The shortcomings of this classification, *e.g.* its lack of further taxonomic considerations, prompted other classification principles to be proposed. Unfortunately, these efforts to bring order only resulted in several parallel nomenclatures and, as a result, many loci have non-conforming and confusing names, as well as several synonymic designations. A more precise way to add specificity is to mention which chromosome the locus is on and exactly which nucleotide positions it occupies in the human reference genome, *e.g.*, HERV-K119 occupies nucleotides 58,721,242–58,730,698 of chromosome 12.

In this review, we focus only on those families that have been proposed to be of potential relevance in human autoimmunity: primarily the “Human MMTV-like 2” (HML-2) subgroup of the Class II HERV-K (26) and the Class I provirus HERV-E (27). We would postulate, however, that individual HERVs that may be detrimental to our health could belong to any family. At the same time, we find it more likely that the most recently incorporated HERVs, which have retained much of their original features and still can produce virions (albeit all with reduced infectivity), are more likely to cause immune disorders resembling chronic viral infections than the older HERVs, which often are incomplete, and have been “domesticated” by frame-shifts, point-mutations, and stop codons. We accept, of course, that older HERVs may have acquired new properties by stochastic mutations and thereby gained the ability to drive unique pathologies unrelated to the mechanisms of typical antiviral immunity.

### HERV-K (HML-2)—The Youngest and Most Intact HERV Family

Although the now (presumably) extinct free retrovirus that gave rise to the HERV-K (HML-2) provirus family (26) repeatedly infected our ancestors for tens of millions of years, we will probably never know what kind of disease it caused at that time. What we can conclude using computational tools from the ~120 genomic loci still present today is that HERV-K(HML-2) first entered our ancestral early hominin germline genome over 30 million years ago (28) and then continued to insert again and again into our germline genome until very recently in evolutionary time (29). Many other retroviruses stopped incorporating into our germline much earlier. Obviously, the potentially vast numbers of infections that did not result in germline insertions are invisible to us today, even if they likely were important for the life-cycle and spread of the virus. Hence, the HERV-K provirus loci in our genome represent a vast underestimate of the number of times the free virus infected and perhaps profoundly affected our ancestors. The insertional polymorphisms (*i.e.* only some people have some of them) (30–32) and the polymorphic deletions (33) observed in human populations today reveal that infections probably continued until times when modern humans were more numerous and had spread out over larger geographical areas in the last 50–70,000 years. Exogenous HML-2 appears to have infected gorillas relatively recently as well (34).

The age of a HERV-K locus (*i.e.* the time since germline integration) can be estimated from the fact that the single LTR in the circular retroviral RNA genome is reverse-transcribed twice, resulting in two identical LTR copies, one in each end of the resulting genomic provirus (**Figure 1**). Since there is no evolutionary pressure to maintain these sequences, they are assumed to be subject to stochastic mutations at the standard background rate of approximately 0.5 x 10^-9^ per base-pair per year. This “molecular clock” obviously can only be applied to HERV-K loci that have retained both LTRs. Based on this logic, an alignment of the seven youngest HERV-K proviruses was used to deduce *in silico* what the sequence of the original infectious retroviruses most likely was. Albeit not necessarily 100% correct, the resulting sequence gave rise to a fully infectious retrovirus, termed HERV-K Phoenix (35), which has been studied for its tropism, cellular receptors, maturation, ultrastructure by electron microscopy, and ability to reverse transcribe and insert its cDNA into the genome of host cells (35).

The most recent human insertions of the HERV-K (HML-2) subfamily, *e.g.*, HERV-K113 at chromosome 19p12 (36), are also intact enough to produce virions (37), albeit with poor infectivity. Other full-length HERV-Ks with intact open reading-frames are HERV-K108a (at 7p22.1), HERV-K115 (8p23.1), HERV-K118 (at 11q22.1), and HERV-K119 (at 12q14.1). Another seemingly intact HERV-K provirus is located at Xp21.33 in approximately 2% of people, most of whom are of African ancestry (31). HERV-K113 and HERV-K115 are also insertionally polymorphic and exist in 15%–30% of modern humans.

To the extent that we know, these youngest loci are transcriptionally silenced in healthy individuals by extensive DNA methylation and other epigenetic mechanisms, as one would assume for potentially dangerous loci. A consequence of this is that they probably remain silent during the development of T and B cell antigen receptor repertoires in early life, resulting in weak immunological tolerance against the proteins that they can encode. If this indeed is true, aberrant expression of these proteins would likely provoke both cellular and humoral immunity (38). There is supporting evidence for this assumption: increased transcription in malignancies of the breast (39) and prostate (40), and in HIV infected individuals (38, 41–45), leads to both (auto)antibodies against HERV-K proteins and HERV-K-specific T cells. Increased levels of HERV-K transcripts have also been detected in rheumatoid arthritis (RA) blood and synovial tissue (46, 47). The resulting immune response is discussed in more detail in section *Autoantibodies Against Retroviral Proteins in Autoimmunity*.

From the perspective of autoimmune diseases like RA and SLE, which are strongly female-biased, it is interesting to note that the 5’ LTR of intact HERV-K loci contain many binding motifs for estrogen- and progesterone-regulated transcription factors. Indeed, these hormones can upregulate transcription many-fold (48). We have replicated this finding (unpublished). HERV-K transcription is also increased by cigarette smoking (49, 50), another risk factor for RA (51, 52).

### HERV-E and Other HERVs of Potential Significance

A body of literature describes findings of increased expression of HERV-E in autoimmune disease, particularly SLE (53), as well as the presence of autoantibodies against HERV-E proteins, such as p30 encoded by its *gag* gene (54, 55). HERV-E derived Env protein can be detected in psoriatic skin (56). Compared to healthy individuals, HERV-E mRNA is reportedly increased in T cells from SLE patients, its LTR is hypomethylated, and further expression can be induced by demethylating agents and UV irradiation (57, 58). It has been proposed that autoantibodies against p30 cross-react with class I HLA (55).

A detailed survey of transcripts derived from over 8,000 retroviral sequences in our genome by Akiko Iwasaki and her team (59) found that a large portion of HERV loci are transcribed in transformed cell lines and many also in cells from patients with SLE. Compared to healthy controls, a number of transcripts were more, and a few less, abundant in SLE patients. The overexpressed loci included several HERV-K, HERV-E, HERV-W, and ERV3 loci. The use of computational tools, such as ERVmap designed by these authors (59), or others (60), have begun to uncover the full complexity of this topic. Case in point: the number of spliced and processed mRNAs derived from all the HERVs theoretically rival those of the traditional genes in numbers. If one also includes all retrotransposon transcripts, which sometimes are derived from in intronic or 5’ and 3’ UTRs of protein-coding genes, the overlap and complexity becomes truly challenging. An important question is which of all these retroviral and retrotransposon transcripts are, in fact, translated into polypeptides that may have consequences for health and disease? Do retroelement transcripts matter if they are not translated? What are the consequences of the production of various retroelement-encoded polypeptides? How might the numerous single nucleotide polymorphisms and other types of polymorphisms within HERVs and retrotransposons affect human health?

### Non-LTR Retrotransposons—LINE-1 and Alu Elements

**Figure 2:**
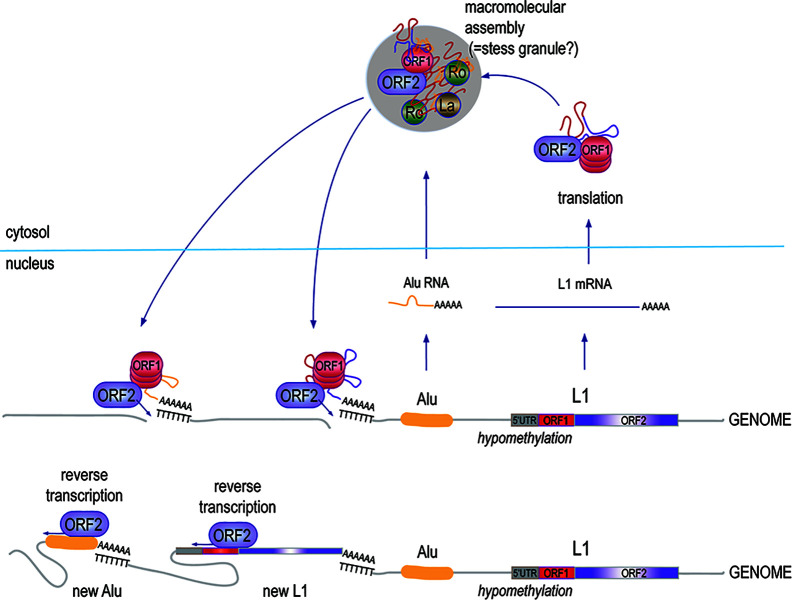
The biology and replication cycle of the L1 element. Approximately, 500,000 copies of the L1 element, most of them truncated and mutated, exist in the human genome across all chromosomes, both within introns of protein-coding genes and in intergenic regions. Transcripts of the few 6-kb full-length L1 loci that retain intact open-reading frames are translated into the p40/ORF1p and p149/ORF2p proteins, which assemble in approximately a 20:1 stoichiometry into complexes with high affinity for RNA, particularly L1 mRNA, as well as Alu RNA. The complexes also include at least 10 other proteins, including Ro60 and La. Under permissive conditions, the RT activity of ORF2p makes a DNA copy of the associated RNA and inserts it into the genome, resulting in a new L1 element, a new Alu element, or a new pseudogene of an accidentally captured other mRNA.

The L1 element (61) appears to represent a remnant of an ancient retrovirus that retained, or later acquired, a degree of autonomy through the conservation of a primordial RT, which endows it with the ability to transpose without having to leave the host cell. This mechanism has been extraordinarily successful over evolutionary time and L1 sequences now occupy 17% of our genome (12, 62–64). While most of the ~500,000 L1 copies are mutated and truncated, some ~180 copies are seemingly intact and a handful of them remain fully active today (65), *i.e.*, they continue to retrotranspose by the L1 “copy-and-paste” mechanism (**Figure 2**), occasionally disrupting genes or regulatory regions by novel insertions (66).

Full-length L1 is a 6-kb sequence with two open reading frames (ORF1 and ORF2) that encode for two proteins: the 40-kDa RNA binding ORF1p protein, and the 149-kDa ORF2p, which has both reverse transcriptase (RT) and endonuclease activities. Retrotransposition occurs through a “copy-and-past” mechanism, where the primary transcript is captured by ORF1p, and is then reverse-transcribed by the RT domain of ORF2p primed by a nick made by the endonuclease domain of ORF2p in a genomic poly-T sequence, where the 3’ poly-A of the L1 RNA can anneal. The DNA repair machinery then patches up the 5’ end of the new insert. From time to time, ORF1p and ORF2p grab the wrong RNA, resulting in the reverse transcription and genomic insertion of an Alu element (67) or a spliced mRNA giving rise to an intron-less pseudogene (63). Even if most pseudogenes are inactive, this mechanism may have created genomic diversity and new material for natural selection to work with. Similarly, ORF2p-mediated reverse-transcription and insertion of a primary (un-spliced) mRNA would result in gene duplication; this may be how gene families were created over evolutionary time.

The RT of L1 ORF2p shows clear, but relatively distant, homology with the RT of the HERVs, suggesting that they all originate from a common ancestral RT. The L1 RT is closer in homology to the RT of hepatitis B virus, a “para-retrovirus”, that uses reverse transcription to generate a circular DNA that is not integrated into the host genome. Insects, like *Drosophila*, have retrotransposons (68). Yeast also have retrotransposons similar to L1, called Ty3 (69), as do baculoviruses (70), and prokaryotes in the form of reverse-transcribing bacteriophage (71). Retrotransposons in plants (72) have been called “engines of evolution” (73). All of these examples attest to the truly old roots of these sequence elements.

There is evidence that L1 loci are transcriptionally active in SLE patients (74–76). This appears to correlate with a global decrease in DNA methylation, which is well documented in SLE (77, 78) and likely relates to the decreased expression of DNA methylases DNMT1 and DNMT3a (79, 80). Demethylating agents like 5-aza-2’deoxycytidine (81) also cause a dramatic upregulation of L1 and Alu element transcription in lymphocytes (82). In addition, transfer of 5-aza-2’deoxycytidine-treated T cells into healthy mice results in an SLE-like disease (83). The drugs that can induce “drug-induced lupus”, notably hydralazine and procainamide, are also demethylating agents (84). Other known triggers of lupus flares, like UV light, oxidative stress, inflammation and exogenous viruses also induce genomic hypomethylation (85, 86). L1 transcription can also be stimulated by female hormones, which further supports the notable female predilection of SLE and RA.

Among the many reasons to suspect that L1 plays a role in autoimmune disease is the observation that ORF1p resides mostly in macromolecular assemblies (**Figure 2**) that have been proposed to be stress granules (87) together with L1 RNA and other RNA-binding proteins (88–91), such as Ro60, La (92), and U1 small nuclear ribonucleoprotein of 70 kDa (88), all well-established autoantigens in SLE and related diseases. The protein complexes also contain ORF2p and perhaps newly synthesized DNA made by its RT activity. If released from broken or dying cells, such protein complexes containing two apparently immunogenic proteins as well as both RNA and DNA would likely be of great interest to the immune system.

## Defense Mechanisms Against Retroviruses and Retrotransposons: Our Original Immunity

In support of the notion that incoming integrating retroviral genomes and autonomously retrotransposing sequences have posed serious threats to the integrity of host genomes since the early days of cellular life (despite also contributing positively to evolution), an elaborate set of defense mechanisms against retroviruses and retroelements are present in all cells (93–98). Many of these mechanisms were discovered in the course of HIV research as “restriction factors”. It also appears that many of these mechanisms continue to be critically important for human health. Conversely, many prevalent diseases, including cancer and autoimmunity, may be related to incomplete function of these mechanisms (61, 99).

### All Roads Lead to Type I IFNs

Type I IFNs are a central hub of antiviral immunity (100). Therefore, it is not surprising that many of the defense mechanisms that cells use against retroviruses and retrotransposons also center on the induction of type I IFNs. Nevertheless, one should keep in mind that many defense mechanisms also have direct anti-viral functions and that many induce other pathways too. Interferons often play an amplifying role and increase the expression of these defenses in a positive feedback loop.

The main threat of a virus is its RNA or DNA genome, which will hijack the cellular biosynthetic machinery for its own replication and virion production, with detrimental and often lethal consequences for the host cell. Even more alarming, retroviruses will reverse transcribe their RNA genome and insert the resulting DNA into the host genome as a permanent provirus. To counteract these ancient foes, evolution has produced several cellular mechanisms for the detection of non-self RNA and DNA (11). Five principal pathways operate in the cytosol and on the cytosolic surface of intracellular organelles: the DNA-sensor “cyclic GMP-AMP synthase” (cGAS) (101), the RNA sensors “retinoic acid-inducible gene I” (RIG-I) (102) and “melanoma differentiation-associated gene 5” (MDA5) (102–104), and the two kinases “protein kinase RNA-activated” (PKR) (105, 106) and DNA-activated protein kinase (DNA-PK). A sixth pathway responds to extracellular DNA or RNA brought into the cell by receptor-mediated endocytosis and is initiated by Toll-like receptors (TLRs) 3, 7, 8, and 9 in the endosome. A mechanism for cross-talk of the extracellular and intracellular sensing pathways consists of the transporter protein SIDT2 (107), which channels dsRNA through the endosome membrane into the cytosol, where it can trigger MDA5. There are several additional recently discovered DNA and RNA sensors, such as DDX1, 21, 36 and 41, IFI16, and Aim2 (108). All these pathways primarily promote type I IFN production through activation of IRF3 and related transcription factors. Some also activate signaling pathways that lead to the production of other cytokines. The resulting type I IFNs are secreted, bind to the type I IFN receptor, and signal through the JAK/STAT pathways to upregulate the expression of proteins with direct anti-viral activity, including nucleases, helicases, chaperones, and many of the sensors and their adapters and signaling proteins (100). Another important effect is the upregulation of MHC molecules to facilitate the recognition of the virally infected cell by cytotoxic T cells.

Most patients with SLE (or related diseases) have elevated levels of type I IFNs (3, 4, 109), which is best detected as the high expression of IFN-stimulated genes (ISGs), now referred to as the “IFN signature” and seen in 70-90% of SLE patient populations world-wide (5, 110–113), as well as in patients with pSS (10, 113), systemic sclerosis (114, 115), polymyositis (PM) and DM (7, 116), and in a small subset of rheumatoid arthritis (RA) (117, 118). The elevated IFNs include not only IFNα and IFNβ, but also the less known IFNε, IFNκ, and IFNω, as well as type II IFN (IFN*γ*) (10), and type III IFNs (IFNγ1, IFNγ2, and IFNγ3) (119), which collectively appear to play an important role in pathogenesis (2, 120, 121). Type I and III IFNs are functionally overlapping (all genes induced by type III IFNs are also induced by type I IFNs), but IFN*γ* is instrumental in a distinct aspect of the immune system, namely the activation of T helper 1 cells, cytotoxic T cells, natural killer (NK) cells, and other elements of a general immune response. Nevertheless, some 900 of the 1,300 ISGs induced by IFN*γ* are also induced by type I IFNs, which induces a total of over 1,500 ISGs, suggesting much overlap in downstream consequences.

Type I IFNs have a spectrum of effects on the immune system and beyond, particularly upregulating numerous aspects of anti-viral defense. They stimulate emergency myelopoiesis (122), monocyte differentiation into myeloid dendritic cells (123, 124), antigen presentation, cytotoxic T cell differentiation (125), and B cell differentiation into plasma cells (126). These hallmarks of anti-viral immunity also characterize SLE and other autoimmune conditions.

### DNases, RNases, and Aicardi-Goutières Syndrome

To neutralize dangerous DNA or RNA, cells express a number of DNases and RNases, the function of which also prevent untimely triggering of DNA and RNA sensors. Remarkable insights into the dynamic biology behind these processes was gleaned from studies of the monogenic disease known as Aicardi-Goutières syndrome (AGS) (127–132), in which loss-of-function mutations in any one of a number of enzymes lead to constitutively high production of type I interferons (IFNs), neurological deficits due to IFN toxicity, and autoimmunity that resembles SLE very much. Loss of the cytosolic DNase *TREX1* (99, 129, 133, 134) causes the accumulation of non-chromosomal DNA made by L1 ORF2p (135, 136), while mutations in any of the three subunits of *RNASEH2* (129, 132) cause the accumulation of DNA : RNA heteroduplexes made by ongoing reverse transcription (132). Another AGS gene, *SAMHD1* (137, 138), directly counteracts reverse transcription by dephosphorylating the required deoxy-nucleotide triphosphates. Together, these defects show that IFN-driving aberrant DNA apparently results from reverse transcription of cellular RNAs at a surprisingly high spontaneous rate. The only cellular enzyme capable of this reverse transcription is the ORF2p of L1, which is a highly efficient RT (61, 139, 140). IFN production (141) triggered by L1 can use many cellular RNA templates, including its own mRNA (63, 64) or Alu transcripts, to generate DNA species that drive the interferon production pathway. This mechanism also operates in cellular senescence (142).

In a mouse model of AGS, the *Trex1* ^-/-^ mouse (99), the animals develop a systemic inflammation with immune cell infiltrates in many organs and they die early from a severe carditis. These animals can be rescued from death by treatment with the RT inhibitors tenofovir plus nevirapine (143), indicating that reverse transcription is a key step in the pathogenesis of systemic inflammation in this model. However, there is also a published paper refuting these data (144). More importantly, the IFN signature can be reduced substantially in AGS patients by RT inhibitors used for the treatment of HIV (145).

### Factors That Reduce Retrovirus Infectivity and Retrotransposition

The fact that the vast majority of HERVs and retrotransposons have been rendered largely inactive and/or harmless (to the best of our knowledge) attests to the power of the spectrum of defensive mechanisms employed by cells both acutely and over evolutionary time. The default acute mechanism employed by cells to silence unwanted or dangerous genes is the modification of deoxy-cytosine in DNA by methylation. This modification also facilitates the addition of suppressive histone H3 K4-dimethyl marks to keep these loci transcriptionally silent. In this context, it is interesting to note that many of the drugs notorious for causing “drug-induced lupus”, such as hydralazine and procainamide, are demethylating agents. Experimentally, 5-aza-deoxycytosine can also be used to reduce the methylation of the genome. This also causes an increase in the expression of L1 and many HERVs. Ultraviolet light (UVB) also reduces genomic methylation, likely with the same de-repression of retroelement transcription. UVB is also a well-recognized trigger of lupus flares.

A good example of retrotransposon control is seen with the large number of interrupted retrotranspositions of L1 in which the reverse transcription was terminated before it reached the 5’ end. As a result of this, many L1 copies lack portions of ORF1 or the 5’UTR regulatory region and cannot retrotranspose. Many of the mutations seen in HERVs and L1 may have been deliberately introduced by the APOBEC family (146) of IFN-inducible cytidine deaminases, which recognize viral or retroviral sequences and rapidly introduce mutations into them (147). This mechanism has been shown to be effective at reducing the virulence of new retroviruses (148, 149) and the ability of retrotransposons to replicate (150).

Another example is Moloney leukemia virus 10 (MOV10), an ATP-dependent helicase that unwinds L1 RNA during reverse transcription to reduce the retrotransposition (151–153) process in a somewhat unclear manner (95). It also participates in the defense against retroviruses (154). MOV10 is located in the macromolecular complex of RNA-binding proteins that also includes L1 ORF1p and ORF2p, as well as SLE autoantigens Ro60 and La (89–91).

There are numerous additional cellular mechanisms to counteract each step of the retrovirus life cycle and the retrotransposition of repetitive elements (155). Many of these mechanism also serve as defenses against other types of viruses and many of them were uncovered in the course of HIV research (98). It is presently not known if any of these mechanisms are compromised in patients with SLE or other autoimmune conditions.

### RNA Interference, Argonaute, Piwi, and Other Nucleic Acid-Based Defenses

Many prokaryotes employ an interesting defense mechanism in which short pieces of the genetic material of past pathogens are kept in a region of the genome to serve as recognition modules for the defense against reinfection. This mechanism (known as CRISPR/Cas9) has an RNA-based counterpart in eukaryotes, including humans, in the form of RNA interference mechanisms that utilize retrotransposon-derived miRNAs, piRNAs, and potentially antisense transcripts from HERVs. Particularly, the piRNA/Piwi pathway appears to be important for protecting the integrity of the germline genome (156). One of the effectors of these still incompletely understood pathways is Z-DNA binding protein-1 (ZBP-1) (157), also known as DAI, which binds dsRNA or DNA that adopt the Z-conformation. ZBP-1 then activates both the IRF3 pathway for type I IFN production and the RIP3 kinase pathway that triggers cell death by necroptosis (158). The relevance of ZBP-1 in Crohn’s disease (159) and other inflammatory conditions (158) was recently demonstrated.

## Molecular Mechanisms by Which Endogenous Retroviruses and Retrotransposons Could Cause Immune Pathology Leading to Autoimmunity

A number of mechanisms have been proposed over the years for how HERVs or L1 could cause diseases like cancer and autoimmunity. In cancer, it is thought that some combination of active retrotransposition catalyzed by L1 ORF2p (160), genomic recombinations caused by highly similar repetitive sequences (*e.g*., LTRs), or promoter/enhancer effects of HERV LTRs or L1 5’ UTRs can create gross chromosomal abnormalities, tumor suppressor disruptions, and loss of normal transcriptional control. In autoimmunity, other aspects of HERV and L1 biology are probably more relevant.

It should be stated upfront that despite the many genetic, experimental, and supportive correlative findings, it is still possible that none of the myriad of HERV and L1 sequences that constitute at least 25% of our genome (close to 40% if one includes Alu elements and other SINEs) have any role at all in autoimmunity because they have been sufficiently “domesticated” and have lost their immunogenicity and potential to raise an anti-viral response or to skew biological processes in any meaningful way. That said, there are many plausible aspects to the general hypothesis that HERVs and/or L1 can promote or even trigger autoimmunity (17, 161, 162). We believe that the vast majority of retroelements are harmless, some even beneficial (17), but that a select few are dangerous and participate in the pathogenesis of common autoimmune diseases like SLE or RA by mechanisms that are discussed below.

### Autoantibodies Against Retroviral Proteins in Autoimmunity

In the 1990s, a number of researchers made the surprising discovery that serum immunoglobulins from patients with RA, SLE, or other autoimmune diseases, reacted with Human Immunodeficiency Virus (HIV) proteins, *e.g.*, p24 of the HIV capsid (163–166), even if these patients had never encountered the virus. Such HIV-reactive antibodies were found in exceedingly few healthy subjects, but reportedly in up to 60% of RA patients. A likely answer to this conundrum was provided by the subsequent discovery (167) that endogenous retroviruses in the human genome, particularly HERV-K (168), are transcriptionally activated in some RA patients (46, 169). This raised the possibility that HIV-reactive antibodies in patients are, in fact, antibodies against HERV proteins that have a sufficient degree of sequence homology with HIV proteins. Indeed, two papers (170, 171) reported that 16% of RA patients have antibodies against an epitope in the HERV-K envelope protein (amino acids 19-37). It should be noted that the percentage of positive patients in the earlier papers was higher, presumably because the tested antigens were full-length proteins in their native state, while later papers mainly used selected peptides and therefore may have missed many autoantibodies.

We have replicated the detection of elevated IgG autoantibodies against HERV-K Env proteins (not peptides) in RA patients (submitted for publication). These antibodies were also present in pediatric patients with juvenile idiopathic arthritis (JIA), and they were higher in smokers than in non-smokers. Anti-Env antibodies were also detected in some control (i.e. non-RA) individuals, in SLE patients (172), and in patients with breast cancer (173, 174) and other hormonally driven cancers. Several HERV-K loci are reportedly transcriptionally active in these cancers (175–177), perhaps through the action of sex hormones on the HERV-K LTRs (48). Notably, RA is a female-biased disease with a 4:1 female-to-male ratio. Autoantibodies have also been reported against proteins of HERV-E, particularly the Gag protein of HERV-E clone 4-1 (178). We would not be surprised if it was found that patients have autoantibodies against additional HERV proteins.

#### Could Autoantibodies Against Retroviral Proteins Be Directly Pathogenic?

The presence of anti-HERV autoantibodies in patients with autoimmune diseases prompted many researchers to wonder if HERVs play a role in autoimmunity. One proposed mechanism to connect these autoantibodies to autoimmune disease was the hypothesis of “molecular mimicry”, postulating that the relevant epitopes for these anti-HERV autoantibodies may be sufficiently similar to amino acid motifs in self-proteins to cross-react with such *bona fide* autoantigens and result in autoimmunity. In our view, this hypothesis (which has also been proposed for exogenous viruses) seems rather unlikely and it is not supported by patient-based data. While a few instances of three to four identical amino acid residues can be found in HERV proteins and proteins like collagen or IgG, these were not shown to be epitopes for autoantibodies. This hypothesis also assumes that retroelement proteins are not “self”, in contrast to proteins encoded by traditional genes, and that humoral or cellular immunity only against the latter could be pathogenic. In addition, central tolerance against the relevant epitopes in traditional self-proteins should automatically also prevent the same sequence from being immunogenic when present in a different class of self-proteins, *e.g.*, HERVs.

**Figure 3:**
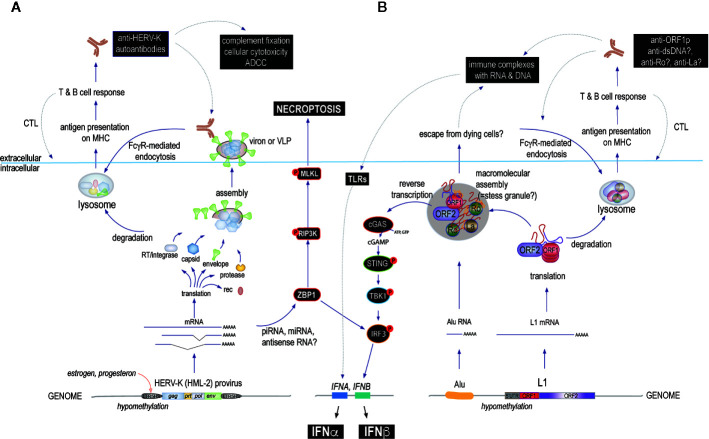
The main proposed mechanisms by which HERVs and L1s may cause immune responses that could escalate to autoimmune disease if they become chronic or recurring. **(A)** starting at the bottom (intracellularly): environmental or internal factors first reduce the suppressive DNA methylation of 5’ LTRs allowing transcription factors such as those regulated by female hormones to actively transcribe one or several HERV loci. The resulting transcripts are spliced and processed and some of them are translated into proteins, while others can associate with antisense RNA, small interfering RNAs of the miRNA or piRNA classes, or form internal loop structures that are recognized by ZBP-1 (or other sensors or RNase-based enzymes), which can signal through IRF3 to IFN production or, alternatively, *via* the RIP3 kinase pathway to cell death by necroptosis. HERV proteins can assemble into more or less complete virions that may remain exposed on the cell surface or even bud off as mature virions. These proteins can be degraded and processed for antigen-presentation on class I or II MHC molecules to activate T cells and B cells to generate both cellular and humoral immunity. Both arms target cells expressing the relevant HERV for immune attack. **(B)** L1 may drive a similar immune response, except that the ORF2p RT can generate intracellular DNA to directly trigger cGAS or other DNA sensors resulting in type I IFN production (primarily IFNβ). Another unique feature of L1 biology is the assembly of the two L1 proteins with other RNA binding proteins, many of which are well-known autoantigens, into aggregates that also contain L1 RNA and other RNA species, such as Alu element RNA or processed mRNAs. These bodies may also contain DNA newly synthesized by the ORF2p RT and hence will even more resemble virions if they are released from dying cells. This would result in an immune response and autoantibodies against several of their components, such as Ro60, in addition to the L1 proteins themselves. Lastly, such autoantibodies would further promote the uptake of L1 protein/RNA/DNA complexes by Fc*γ*R-expressing immune cells, including plasmacytoid dendritic cells and further stimulate type I IFN production (primarily IFNα) through TLR pathways. In all these scenarios, the repeated boosting of anti-HERV or anti-L1 immune responses would lead to increasingly powerful immune-mediated destruction of the cells that express them. Disease-relevant pathways are indicated with black boxes.

Another simple hypothesis focuses on the plasma membrane location of Env proteins. When expressed, these transmembrane glycoproteins cluster into microdomains together with intracellular Gag to form virions that eventually bud off to leave the host cell. During this time, autoantibodies against Env would be predicted to bind the exposed Env with potential consequences like complement fixation or antibody-dependent cellular cytotoxicity (**Figure 3**). In both cases, the cells expressing Env can be killed under circumstances that would be pro-inflammatory. In this scenario, inflammation would follow the same pattern as in antiviral immunity, except that the offending virus is a HERV and the response would be that of autoimmunity. Since the HERV in question is irrevocably fixed in the host genome, it cannot be eradicated by the immune response and any cell that subsequently expresses Env would be treated as a virally re-infected cell by a recall immune response. This could well result in a pattern akin to what we see in clinically relevant autoimmune disorders.

#### Could Protein Citrullination be Linked to Retroviral Proteins?

Among the autoimmune diseases, RA is unique in that post-translational deimination of arginine residues, also known as citrullination, plays an important role in creating autoantigens. While a low amount of citrullination is a part of normal physiology, much elevated levels of citrullination of proteins that perhaps never are citrullinated at all in healthy individuals can be induced by a process termed “lethal hypercitrullination” or “leukotoxic hypercitrullination” (179, 180). This reaction is induced by any agent that creates pores in the plasma membrane that are large enough to allow Ca^2+^ to rush into cells, such as the membrane-attack complex of activated complement, polymerized perforin from cytotoxic T cells or NK cells, or certain bacterial toxins like α-toxin from *Staphylococcus* *aureus*. Among immune cells, neutrophils are the most prone to undergo a strong hypercitrullination reaction (180). Hence, if the neutrophils express Env in an RA patient with anti-Env autoantibodies, these cells could well be killed by complement or by Env-specific cytotoxic T cells. This hypothetical model of neutrophil killing and hypercitrullination linked to anti-HERV immunity is under investigation in our laboratory.

### Autoantibodies Against Retrotransposon Proteins and Associated Proteins

We recently reported that SLE patients also frequently have autoantibodies against the L1-encoded ORF1p protein (181), which is physically associated with Ro, La, snRNP70, and other well-known SLE autoantigens (87, 88, 91, 182) together with RNA in macromolecular assemblies (which may be stress-granules). Indeed, SLE autoantibodies recognized several other proteins in purified ORF1p-containing complexes (181). Furthermore, anti-ORF1p titers correlated with SLE disease activity, lupus nephritis, anti-dsDNA levels, and complement consumption (181). They were also present in pediatric lupus patients with newly diagnosed disease (our unpublished observation). In contrast, RA patients were negative for anti-ORF1p antibodies with only sporadic exceptions.

At this time, we do not know whether anti-ORF1p autoantibodies are pathogenic *per se*. Because ORF1p is intracellular, and hence out of reach for extracellular antibodies, we are inclined to believe that they do not contribute directly to pathology in SLE and merely reflect the expression of immunogenic ORF1p. However, it is likely that they form immune complexes with extracellular ORF1p, which could escape from dying or broken cells (**Figure 3**). If so, intact ORF1p likely would have bound RNA and exist in a complex with Ro60, La, and other proteins that it associates with in the cells. Such immune complexes could induce type I IFNs upon internalization by plasmacytoid dendritic cells and could promote antibody responses against all the proteins and nucleic acids in these immune complexes. In support of this possibility, isolated anti-Ro60 immune complexes from SLE patients were shown to contain RNA from both Alu elements and L1 (182).

### Direct Pathogenic Effects of Proteins Encoded by HERVs and Retrotransposons

After the initial discovery of autoantibodies reactive against HERV-encoded polypeptides in the 1990s, researchers began to search for the corresponding mRNAs and proteins in a variety of cells and tissues, such as RA synovium (183, 184), initially using degenerate primers and little insight into the shear multitude of retroviral and retrotransposon sequences in our genome. With the sequencing of the human genome (12), the complexity of the issue became more obvious. To this day, there are relatively few commercially available reagents to detect HERV or L1 proteins and only a limited number of computational tools to analyze retroelement transcription profiles in RNA-Seq data sets. Furthermore, it is now clear that large numbers of retroelement loci are transcribed even in healthy individuals (59), including many that probably do not encode for proteins or only for short peptides if translated at all.

The presence of autoantibodies against HERV and L1 proteins suggests that they are at least moderately immunogenic. An important unanswered question is to what extent retroelement-encoded polypeptides are expressed in the thymus and bone marrow during T and B cell antigen receptor repertoire selection and the formation of central tolerance. From the acute use of extensive DNA methylation to silence the transcription of unwanted and potentially dangerous sequences, we project that the youngest HERVs are least likely there and that tolerance against many of them is weak or absent.

In agreement with this notion, a recent paper (185) demonstrated that pancreatic islets in non-obese diabetic (NOD) mice (but not control mice) release microvesicles, which contain endogenous retroviral Gag and Env proteins, probably in the form of complete or near-complete virions. The NOD mice developed antibodies against these proteins, as well as specific T cells, which caused diabetes when adoptively transferred. Elimination of Gag prevented diseases. These data show that abnormal activation and expression of endogenous retroviruses can trigger an anti-retroviral immune response and autoimmunity (185). This scenario is also depicted on the left side of **Figure 3**.

Besides acting as antigens for the host immune response, the proteins and peptides encoded by HERVs and L1 have other properties that could be relevant (17). For example, retroviral Env and Gag proteins can mediate cell fusion events, while mature and processed Env of HERV-K, which form transmembrane trimers, binds to heparan sulfate-containing surface proteins (186). Hence, aberrant expression of these proteins may cause pathological fusion and adhesion events that could prove problematic. In addition, certain Env portions may have immunomodulatory effects.

The “superantigen” encoded by the *env* gene of HERV-K18, which was incorporated into the human genome 7.8–14.4 million years ago and has accrued a number of amino acid substitutions in its *env* gene, made a big splash in the field when its T cell activating properties and presence in type 1 diabetes patients was first reported in *Cell* in 1997 (187). The proposition was that this protein causes a polyclonal T cell activation, which then leads to autoimmunity. Subsequent papers were not in full agreement (188) and despite subsequent papers finding this “superantigen” expressed in JIA patients (189) and that it is inducible by Herpes viruses (190), the interest in HERV-K18 slowly waned. Nevertheless, the lesson from this specific case is that random mutations may not only reduce the ability of a retroviral component to cause pathology, but may also, by chance, give them new and dangerous properties. Certain sequences in the Env protein also appear to have immunosuppressive properties. How such motifs might act and whether they were important for the life-cycle of free infectious HERV-Ks are not known.

The L1-encoded ORF1p and ORF2p proteins have been detected in samples from patients with SLE or pSS (74) by immunoblotting and immunohistochemistry. ORF1p was present in kidney biopsies from lupus nephritis patients and in salivary gland from pSS patients. This staining coincided with IFNβ in glandular cells and with IFNα in the infiltrating immune cells. As activation of L1 elements in autoimmune patients (74–76) appears to involve demethylation of the 5’ UTR (74, 76, 78), these authors also analyzed the methylation of CpG sites in this regulatory region and found it to be reduced in patients with elevated L1 expression. L1 ORF2p is also present in the ductal cells of salivary gland biopsies from patients with pSS (191).

We have detected ORF1p protein in neutrophils of juvenile and adult SLE patients, including in low-density granulocytes from these patients (submitted for publication). These findings are compatible with the emerging role of the neutrophil as a cell type of interest in SLE pathogenesis.

### Could Transcripts From Proviruses and Retroelements Be Pathogenic?

While primary transcripts from endogenous retroviral or retrotransposon loci are synthesized by the same machinery that transcribes and processes other genes and therefore should be indistinguishable from any other cellular transcripts, two recent papers published in *Nature* (158, 159) showed that they have a propensity to form double-stranded structures, perhaps through transcription of the complimentary strand as well. They found that such dsRNAs are recognized by the host defense protein ZBP-1, which binds both DNA and RNA in their Z-conformation. This binding activates ZBP-1 to trigger type I interferon production through IRF3, as well as activation of the RIP3 kinase pathway leading to cell death by necroptosis. Necroptosis, in turn, is a very immunogenic process and leads to autoimmune disease (158, 159).

Another possibility is that some species of retroelement RNA may have features or motifs that somehow resemble viral RNA and therefore are recognized by the RNA-sensors RIG-I or MDA5 (192), a challenging task given the abundance of cellular RNA species. Antisense sequences (transcripts from the other DNA strand) could provide the answer. The delicate balance between the recognition of self- versus foreign RNA is well illustrated by the *IFIH1* A946T allele, which encodes a variant of MDA5 that enhances anti-viral immunity, but increases the risk of autoimmunity (193, 194).

It is curious that some retroelement RNAs like Alu transcripts need to be edited by adenine deimination catalyzed by ADAR1 to remain harmless (195). In the absence of this editing (*e.g*., loss of ADAR1), these RNAs form double-stranded hairpin loops that trigger the RNA sensors leading to AGS (196). There is also evidence that some 30% of SLE patients have constitutively activated RNA sensors, detectable as an aggregation of the downstream mitochondrial antiviral signaling (MAVS) adaptor protein (197). It is not known what RNA species was responsible for this activation in the patients.

### Reverse Transcriptases, DNA Sensors, and Type I Interferons

A mechanism with clear potential for pathogenicity is the conversion of retroelement RNA into extrachromosomal DNA by reverse transcription. If not rapidly degraded by the DNase TREX1 (or other DNases), such aberrant DNA will trigger DNA sensors like cGAS, which in turn drive the production of type I IFNs. This is apparently what happens constitutively in patients with loss-of-function mutations in *TREX1*. It also appears to occur in at least a subset of SLE patients: the second messenger 2’3’-cyclic-guanosine-adenosine-monophosphate (cGAMP), which is synthesized exclusively by cGAS upon DNA binding, was detected by mass spectrometry in 7 of 30 SLE patients (198). While it may seem that this represents a small portion of SLE patients, it is important to recognize that the data represent a single snap-shot in time for each patient and that cGAMP is a short-lived second messenger present in minute quantities. Thus, it may well be that cGAMP is periodically elevated in many more SLE patients than reported.

While the DNA species that triggers cGAS in SLE patients remains unknown, there are only two likely possibilities, as we have discussed before (11): mitochondrial DNA or DNA synthesized by a cellular RT. There are only three types of RTs in our genome: telomerase (*TERT*), the RTs encoded by the *pol* genes of HERVs, and ORF2p encoded by L1. Telomerase only synthesizes TTAGGG repeats in the ends of our diploid chromosomes using the *TERC* RNA template (199, 200), while retroviral RTs supposedly only convert the RNA genome of an incoming retrovirus to a DNA provirus that is inserted into the genome during acute infection. Hence, L1 ORF2p is the most likely to produce DNA that can trigger type I IFN production through cGAS activation in SLE patients. ORF2p has robust RT activity (139, 140, 201), which is key for retrotransposition (202), and is sensitive to some clinically used RT inhibitors (203, 204).

### Possible Roles of Genomic Alterations Resulting From Retrotransposition in Autoimmunity

Lastly, a unique potential mechanism by which retrotransposons, and perhaps also HERVs, could impact human health is by retrotransposition, *i.e.* by inserting a brand-new reverse-transcribed copy into a new genomic location. This can occur early in embryogenesis (205) when the genome is broadly hypomethylated and extensively transcribed, including retroelements of all classes. During this time, RT activity is high, extrachromosomal DNA is readily detectable, and L1 elements are capable of active retrotransposition (206). In fact, more than a hundred novel genetic diseases have been found to be caused by L1 retrotranspositions into vital genes (207), disrupting their regulation or function. It is also clear that active L1 retrotransposition occurs in certain neurons during development of the central nervous system (208) and that this creates somatic mosaicism of unclear neurological relevance (209). It is also possible that similar L1 retrotranspositions could occur in immune cells to generate T cells with abnormal behavior, leading to autoimmunity. One could imagine that the disruption of the gene for an important negative regulator of immunity, for example in a hematopoietic stem cell, could result in populations of overly reactive T cells. While this is an interesting possibility, there is no evidence of it at this time other than in cancer.

A different type of genomic alterations also occurs in malignant cells, namely the recombination of retroelement loci that have a high degree of sequence identity but located in different chromosomes. The large number of single LTRs (“solo-LTRs”) throughout our genome appear to be the result of such recombinations. Again, there is no evidence for this type of genomic alterations in human autoimmunity, but it might be worthwhile to search for them.

### Malignancy-Related Autoimmunity

An immune response against a tumor involves the recognition of tumor-specific epitopes, which consist of point-mutations in common self-proteins, aberrantly spliced or modified proteins, or proteins that normally are only expressed during early embryonic development (*i.e.* carcinoembryonic antigens). Particularly when the immune response against the tumor is strong and succeeds in eliminating the malignant cells, there is an obvious risk of epitope spreading and further escalation into autoimmunity. There are numerous examples of such “paraneoplastic” syndromes and collateral damage, for example vitiligo (i.e. the killing of normal melanocytes) in patients with melanoma after a successfully boosted anti-tumor immune response.

Carcinogenesis typically involves a broad genomic demethylation and de-repression of many genes, including carcinoembryonic antigens, as well as numerous HERVs and L1s. It therefore seems very likely that proteins encoded by these retroelements serve as tumor-specific antigens. This notion is supported by the presence of anti-HERV-K autoantibodies and HERV-K-specific T cells in breast cancer (173, 174). If this response is successful and eradicates the (pre)malignant cells, the immunological memory of these antigens would readily serve to re-engage the immune system if the same proteins were expressed again in a non-malignant cell type. There is currently no clear evidence that patients with autoimmune disease would have fought off a malignancy prior to developing their autoimmunity, but this possibility should be explored if possible.

## Testing the Hypotheses

Resolving the question of whether HERVs and/or L1 retrotransposons contribute to human autoimmune diseases is a monumental challenge. Ultimately, only significant effects of therapeutic interventions in double-blinded, placebo-controlled human clinical trials will be able to conclusively prove causalities that have been inferred from molecular mechanistic experiments, correlative patient observations, and, perhaps, animal models. Disproving a proposed mechanism is even harder.

Regarding animal models, due to the approximately 80–100 million years of evolutionary distance between mice and humans, there are fundamental differences between our repertoires of retroviruses and retrotransposons. Most of the human HERVs are more recent, and do not exist in the mouse genome. Instead, mice still have many fully competent retroviruses, such as mouse mammary tumor virus (MMTV), which is distantly related to the HERV-K family. Disease models based on MMTV would be difficult to interpret. It is very interesting to note, however, that the autoimmunity-prone strains of mice (e.g. MRL, or NZB) have larger sets of active endogenous retroviruses than other strains. Mice also have numerous L1 insertions, albeit not quite as many as humans.

It will be important to construct a set of plausible molecular hypotheses of pathogenesis and to test them for supportive patient-based evidence, or, conversely, lack thereof. Since many diseases like SLE are clinically heterogeneous, one cannot assume that the molecular mechanisms that underpin them will be the same in every patient. Instead, it might be more productive to start with the assumption that each disease contains two or more “endotypes”, *i.e.* patients with a clinically similar disease, but with distinct molecular underpinnings. This concept is well accepted in oncology and is making inroads in respiratory diseases, where asthma is now understood to contain “eosinophilic asthma”, “Th2-high asthma”, “epithelial asthma”, and “allergen/IgE asthma”, each of which respond well to different targeted therapies. These endotypes of asthma are not possible to distinguish clinically, but require laboratory measurements of eosinophils, cytokines, or IgE to classify and treat.

To advance, and eventually elucidate, the role of HERVs and retrotransposons in human autoimmunity, some major tasks that should be undertaken include:

- A comprehensive cataloguing of the expression of HERVs and retrotransposon transcripts that are differentially expressed in cells and tissues from a range of autoimmune and other diseases, with an emphasis of disease-to-disease comparisons, and a filtering of the data sets by the capacity of transcripts to be translated.
- A broader and more detailed characterization of which proteins encoded by HERVs and L1 that become targets of autoantibodies in patients with different autoimmune diseases.
- The use of high-sensitivity proteomics to determine when and where such proteins are present, including an analysis of whether they are expressed in the thymus.
- The identification of T cell epitopes on retroelement proteins and characterization of the relevant T cells.
- Identification of the exact nature and source of nucleic acid species that drive type I IFNs in patients with type I IFN gene signature.
- An evaluation of the roster of anti-retroelement defense mechanisms to identify disease-related deficiencies or abnormalities in patients.
- A thoughtful selection of testable drug targets based on plausible mechanisms, followed by the development of therapeutic molecules that will enable clinical trials with relevant pharmacodynamic measures (biomarkers) to ensure that the drugs have the desired biological impact before asking if they have therapeutic effects.

## Therapeutic Implications

Currently used therapeutic regimens for autoimmune conditions consist of more or less broadly immunosuppressive drugs, which often provide unsatisfactory efficacy and/or unacceptable safety concerns. The development of more efficacious drugs with more precision and therefore, hopefully, improved safety profiles is very challenging as long as our understanding of the molecular mechanisms that initiate and propagate these diseases is so incomplete. We anticipate that scientific discoveries and breakthroughs in the coming years will open up new avenues for the development of better new drugs (210). If some of the hypotheses discussed in this review have any merit, what might new drug targets look like?

An already tested opportunity is the direct inhibition of type I IFNs, which appear to be instrumental in the pathogenesis of SLE and related diseases (111, 211). These drugs have been both encouraging and disappointing. Anifrolumab, an antibody that blocks the type I IFN receptor (IFNAR1) used by all type I IFNs, met with a statistically significant efficacy in phase 2 clinical trials in SLE (212) and met its primary endpoint in one of two phase 3 trials. In contrast, two different antibodies that block IFNα alone (213), sifalimumab and rontalizumab, were efficacious only in a small subset of SLE patients. Together, these trials reveal that type I IFNs beyond the 13 isotypes of IFNα are important, at least in some patients. Furthermore, although anifrolumab was clinically more efficacious, and neutralized the IFN signature by over 90% in the treated patient population, it did not benefit all the included patients.

Inhibitors of protein kinases that mediate the signaling from IFNAR, such as the JAK-family kinase TYK2, or signaling pathways that lead to type I IFN production, like TBK1, or the necroptosis-inducing RIP3K, could also prove therapeutic even if these pathways are in broader use. Another possibility is to intervene in the biology that produces the nucleic acid species that initiate type I IFN production. Because anti-viral immune responses that include production of type I IFNs also include many additional pathways (although many of them amplified by IFNs), such therapeutics may be more efficacious than IFNAR blockade. Drugs that stimulate DNases or RNases, augment their function, or prevent their negative regulation, or that inhibit the enzymes that produce the offending DNA or RNA, e.g. reverse transcription, could be therapeutic. Indeed, RT inhibitors are efficacious in IFN-driven systemic inflammation observed in the *Trex1* ^-/-^ (a DNase) mouse model of AGS (143) and reduce the IFN signature in patients with AGS (145). RT inhibitors also eliminated all symptoms of the pSS-like diffuse salivary and lacrimal gland inflammation in HIV patients in a small clinical trial (214). It will be interesting to see if RT inhibitors would also benefit SLE patients.

The DNA- or RNA-sensors that are activated by aberrant or excessive nucleic acid species could also be targeted by inhibitors. Indeed, c-GAS inhibitors are under development in a growing number of companies, which is also true for drugs targeting RIG-I or MDA5. An obvious risk with these drugs is that they can compromise anti-viral immunity. This is also true for antibodies that block type I IFNs yet appears to be manageable.

As we learn more about the immunogenicity of retroelement proteins and how various retroelement transcripts act to trigger ZBP-1 or RNA sensors, additional drug targets likely emerge. However, it currently seems that most of the transcriptional regulation and transcript processing of retroelements is mediated by the same cellular factors that regulate and process our traditional protein-coding genes. Retroviral LTRs, for example, use a host of transcription factors that regulate numerous other genes as well. Nevertheless, it is possible that RNA-based therapeutic molecules could be designed that more selectively interfere with pathogenic sequences. This space is still totally unexplored.

## Diagnostic and Prognostic Implications

The heterogeneity in clinical manifestations of autoimmune diseases (215) often make them challenging to accurately diagnose and properly treat, and to determine their prognosis with any precision. This challenge has prompted the development of various high-sensitivity and -specificity clinical and laboratory classification criteria and disease activity indices to help in the management of patients with these diseases. However, while these methods are reasonably objective and helpful in clinical trials, there is wide variability in their applicability owing to the often-seen discordance between laboratory evidence of immunologic activity and actual physical evidence of clinical disease activity.

This discordance between laboratory and clinical disease activity leaves plenty of room for improved or adjunctive diagnostic methods that could help in closing that gap, and perhaps also help to improve the prognostication of the diseases relative to treatment. Given the significant correlations of anti-HERV-K Env antibody with RA disease activity (170, 171) (also our unpublished data) and anti-L1 ORF1p antibody with SLE disease activity (181), the potential for their use as diagnostic and prognostic markers is not too far-fetched. By the same token, with regard to potential surrogate markers for innate immune system activity in disease flares (i.e. type I IFN production in SLE), a similar approach could be undertaken whereby IFN signature gene expression is routinely monitored. However, we understand that these principles are still experimental and thus require more investigations for the validation of their actual clinical utility.

## Author Contributions

TM and KU contributed equally to the writing of this review and share accountability for its content. All authors contributed to the article and approved the submitted version.

## Funding

Our work is supported by NIH grants T32 AR007108 (to KU), R21AR075134 (TM), R01 AR074939 (TM), and R21 AR077266 (TM).

## Conflict of Interest

TM has received consulting fees from Cugene, Kiniksa, Miro Bio, and QiLu Pharmaceuticals, has an ownership share in Amdax, and has received research funding from Gilead Sciences.

The remaining author declares that the research was conducted in the absence of any commercial or financial relationships that could be construed as a potential conflict of interest.

## Glossary

Autoantibody: antibody reactive with an antigen encoded by the host’s genome, including retroelements.
DNA transposon: genomic sequences that can move to new locations in the genome without a reverse-transcription step.
HERV: human endogenous retrovirus, the genomic sequence (in its current form) of an originally free retrovirus that infected our ancestors and inserted its reverse-transcribed genome into the germline of their host. HERVs are inherited in a Mendelian fashion. The HERVs have accumulated genetic alterations, such as point mutations, small or large deletions, insertions, etc in a time-dependent manner.
LTR: long terminal repeat, the approximately 650-bp region of the retroviral genome that exerts transcriptional control. The LTR contains numerous transcription factor binding sites and the transcriptional initiation site, but is normally silenced by DNA methylation and histone modifications and other epigenetic means. There is a single LTR in the circular RNA genome of a free retrovirus, but during reverse-transcription for genomic insertion, the LTR is reverse-transcribed twice, ending up in the 5’ and 3’ ends of the insert.
Retroelement: general term for all genomic sequences that can be transcribed into RNA, reverse-transcribed to DNA, and then reinserted into a new location of the genome. Endogenous retroviruses are LTR-retrotransposons, while other retrotransposons are not.
Retrotransposon: essentially a synonym of retroelement, but is often used for non-LTR retroelements.
Provirus: the genomic cDNA for a retrovirus, endogenous or exogenous.
Solo-LTR: as a result of annealing between the two identical LTRs of a retroviral provirus followed by excision repair, only one LTR sequence is left in the genome. An insertionally polymorphic HERV may be present in the human genome as a provirus, a solo-LTR, or be absent. In the latter case, the location is referred to as “pre-integration” sequence.
Reverse transcriptase: the enzyme that synthesizes a DNA strand complementary to a single-stranded RNA template. Upon completion, an RNase domain of the reverse-transcriptase degrades the RNA template, allowing the reverse transcriptase to synthesize a second complementary DNA strand.
